# Isolation and usage of exosomes in central nervous system diseases

**DOI:** 10.1111/cns.14677

**Published:** 2024-03-18

**Authors:** Wenjing Wang, Hong Sun, Huijuan Duan, Gang Sheng, Na Tian, Dingyi Liu, Zhaogang Sun

**Affiliations:** ^1^ Beijing Chest Hospital Affiliated to Capital Medical University Beijing China; ^2^ Beijing Tuberculosis and Thoracic Tumor Research Institute Beijing China

**Keywords:** biomarker, central nervous system, diagnosis, exosomes, isolation method

## Abstract

**Background:**

Exosomes are vesicles secreted by all types of mammalian cells. They are characterized by a double‐layered lipid membrane structure. They serve as carriers for a plethora of signal molecules, including DNA, RNA, proteins, and lipids. Their unique capability of effortlessly crossing the blood–brain barrier underscores their critical role in the progression of various neurological disorders. This includes, but is not limited to, diseases such as Alzheimer's, Parkinson's, and ischemic stroke. Establishing stable and mature methods for isolating exosomes is a prerequisite for the study of exosomes and their biomedical significance. The extraction technologies of exosomes include differential centrifugation, density gradient centrifugation, size exclusion chromatography, ultrafiltration, polymer coprecipitation, immunoaffinity capture, microfluidic, and so forth. Each extraction technology has its own advantages and disadvantages, and the extraction standards of exosomes have not been unified internationally.

**Aims:**

This review aimed to showcase the recent advancements in exosome isolation techniques and thoroughly compare the advantages and disadvantages of different methods. Furthermore, the significant research progress made in using exosomes for diagnosing and treating central nervous system (CNS) diseases has been emphasized.

**Conclusion:**

The varying isolation methods result in differences in the concentration, purity, and size of exosomes. The efficient separation of exosomes facilitates their widespread application, particularly in the diagnosis and treatment of CNS diseases.

## Graphical abstract

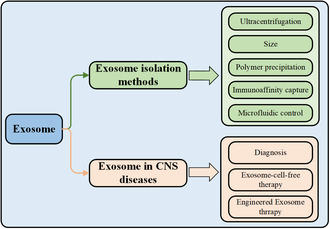

## 1 INTRODUCTION

Extracellular vesicles (EVs) are secreted by various cells and organisms.^1^, ^2^, ^3^, ^4^, ^5^, ^6^, ^7^ They can be categorized into distinct types depending on their size and origin. The main types of EVs are exosomes (50–200 nm), microvesicles (100–1000 nm), apoptotic bodies (50–4000 nm), and prostasomes (40–500 nm).^8^ In particular, exosomes are small, spherical vesicles with a lipid bilayer and have a density ranging from 1.13 to 1.19 g/mL. They were first identified in sheep reticulocyte cells in 1983 and officially named “exosomes” 4 years later by Johnstone.^9^, ^10^, ^11^ Although the term “exosomes” is commonly used, it is recommended that the term “small extracellular vesicles (sEVs)” be substituted to accurately represent the diverse population of EVs.^12^ Exosome biogenesis occurs in two distinct stages: first, membranous vesicles form through inward budding within endosomes, and then these vesicles are released into structures called multivesicular bodies.^13^ Exosomes can transport membranous and cytoplasmic components from their parent cells, including proteins, messenger RNA (mRNA), microRNA (miRNA), long noncoding RNA (LncRNA), lipids, metabolites, and even fragments of DNA.^14^, ^15^ The contents and functions of exosomes from different cells are specific. In the early days, people believed that the exosomes were the “scavengers” in the cells, responsible for carrying the wastes generated by cells.^16^ Afterward, the function of exosomes in the central nervous system (CNS) disorders attracted increasing attention with extensive research on the biological origin, material composition, transport mechanisms, intercellular signaling, and distribution in the body fluids of exosomes. Exosomes participate in intercellular communication^17^, ^18^ and regulate physiological and pathological processes.^19^ They are used for diagnosing CNS disorders^20^ and are also considered ideal candidates for drug delivery systems (DDSs) in the treatment of CNS diseases.^21^ Exosomes, as therapeutic agents or DDSs, offer the following advantages. Being naturally derived from cells, exosomes possess inherent biocompatibility and biodegradability. This reduces the risk of immune rejection or adverse reactions, making them a safer drug delivery option compared with synthetic carriers.^22^, ^23^ Moreover, their lipid bilayer structure not only provides stability but also shields the encapsulated cargo, ensuring efficient delivery to target sites.^24^ Exosomes can be engineered to carry specific targeting molecules on their surface. This feature enables exosomes to selectively deliver therapeutic cargo to the desired cells or tissues.^25^

Given the significance of exosomes in biomedical research, their isolation and purification are critical for comprehending their mechanisms of action and their potential applications in biomedical science. Exosome isolation methods refer to the techniques developed to enhance the concentration and quality of exosomes. Despite the recognized importance of exosomes, accurately characterizing their biological activity largely hinges on the efficiency of diverse exosome separation methods. Furthermore, the current outlook for clinical research on exosomes is not optimistic. The main reasons are as follows^1^: exosome mass production and purification have not yet been developed; and^2^ clinical production of exosomes does not have an international standard. Hence, careful selection of an appropriate isolation method that allows for the efficient and reliable extraction of exosomes from diverse biomaterials and other EVs is essential. Research on different exosome isolation technologies has become a hotspot in recent decades. These technologies can be broadly classified based on their underlying principles of separation, such as ultracentrifugation, size‐based separation, polymer precipitation, immunoaffinity capture, and microfluidic separation, among others.

This study aimed to summarize the current research progress in exosome isolation through typical examples and the application of these isolation methods to the CNS. Subsequently, the characteristics, applications, advantages, and disadvantages of different methods were compared to assist researchers in selecting appropriate exosome isolation techniques. The results might provide a reference for improving the standardized process of exosome extraction and preparation, promoting the clinical transformation and application of exosomes. Finally, we have systematically summarized the application of exosomes in diagnosing and treating common CNS diseases, aims to provide a reference for future research on exosomes as diagnostic biomarkers and therapeutic carriers in CNS diseases.

## 2 ISOLATION OF THE EXOSOMES

**FIGURE 1:**
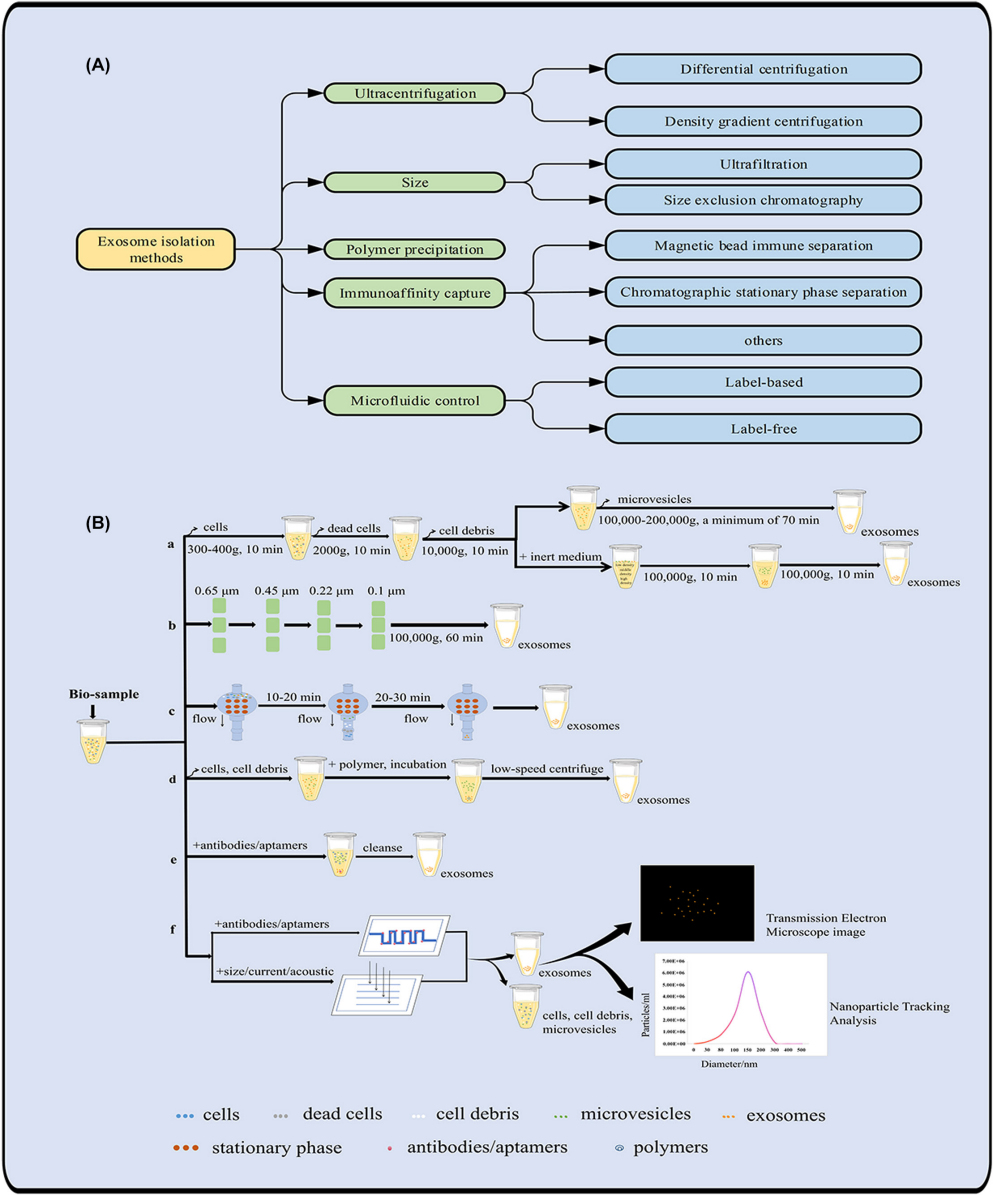
The classification of isolation methods of exosomes and the schematic diagram of their isolation principle. (A) The classification of isolation methods of exosomes. (B) The schematic diagram of exosomes isolation method principle.

**FIGURE 2:**
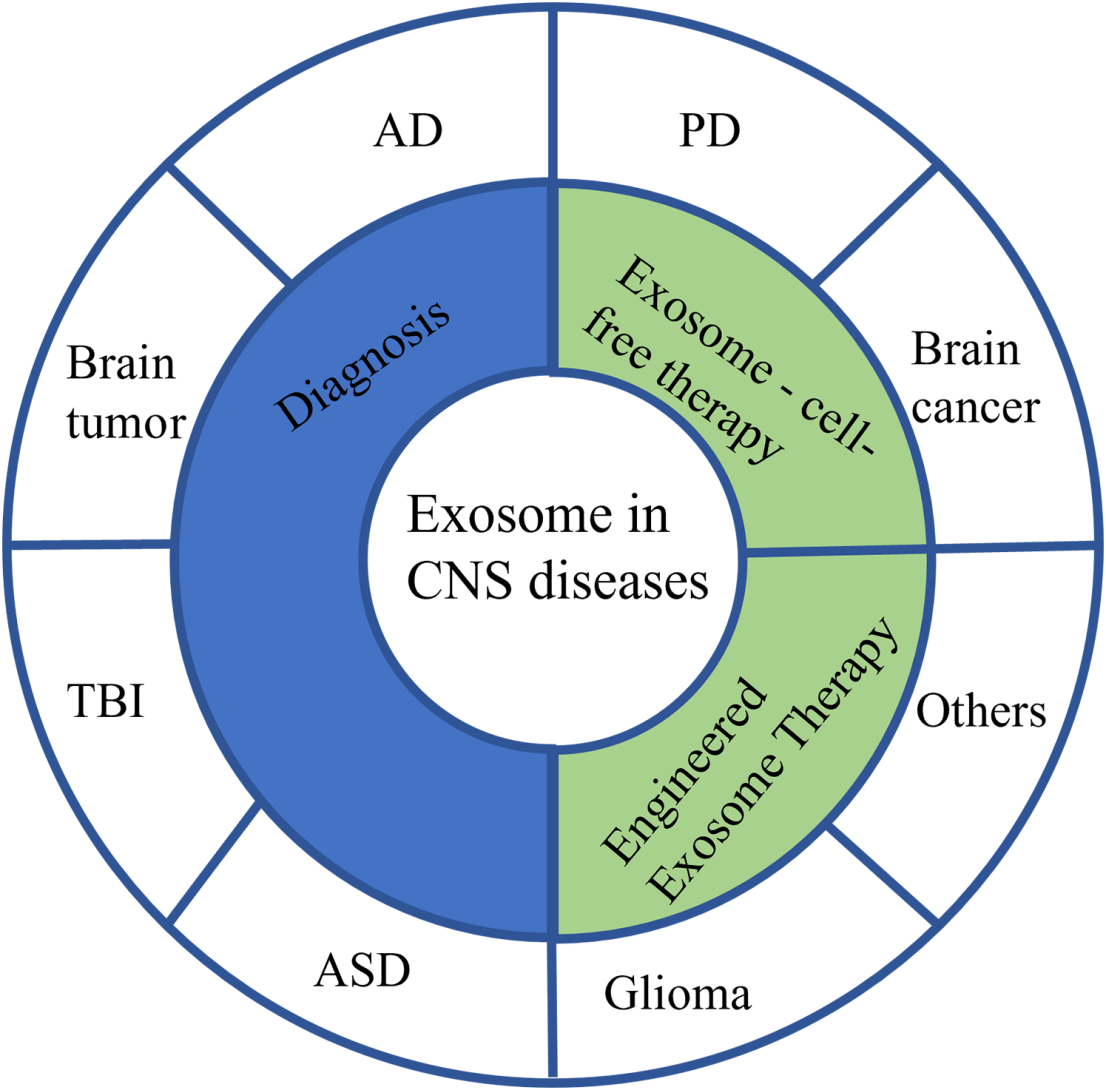
The overview of exosomes in the diagnosis and treatment of CNS diseases.

The isolation and enrichment of exosomes from complex biological components play a vital role in both fundamental research and the advancement of clinical applications. However, the isolation and purification of exosomes from complex biological fluids are challenging due to their small nanoscale size and low buoyant density. Despite the obstacles encountered, researchers have made notable progress in exosome isolation using various techniques. These include ultracentrifugation, ultrafiltration, size exclusion, polymer‐based precipitation, affinity capture, and microfluidic technology, among others (Figure 1A). And, numerous laboratories have successfully isolated exosomes using these diverse approaches.^26^ Nevertheless, the concentration, purity, and size of exosomes vary due to different isolation methods.^27^, ^28^ This section describes exosome isolation based on various isolation principles, with a detailed description of each method (Figure 1B). The benefits and drawbacks of various isolation methods have been compared to aid researchers in choosing suitable techniques for exosome isolation based on their specific conditions and conducting relevant studies (Table 1). Good separation efficiency helps promote the widespread application of EVs, especially in the field of CNS diseases that require breakthroughs in the blood–brain barrier (BBB) (Figure 2).

**TABLE 1:** Comparing various techniques for exosome isolation.

Separation technology	Principle	Advantages	Disadvantages	Application	Refs
Ultracentrifugation	Size and density	Requires a minimum of reagents and expertise, and is suitable for large sample volumes	Time‐consuming, expensive equipment required, lack of specificity in purification, reduction in biological activity, and small quantities of precious samples	Urine, serum, plasma, cerebrospinal fluid, ascites, and cell culture media	29, 30
Density gradient centrifugation	Density	High purity	Time‐consuming and cumbersome steps	Urine, serum, plasma, cerebrospinal fluid, and cell culture media	31, 32, 33, 34, 35
Ultrafiltration	Size and molecular weight	High purity, fast, no need for expensive equipment, and large‐scale use	Low yield, pore clogging, easily deformed or cracked	Urine, serum, plasma, and cell culture media	36, 37, 38
Size exclusion chromatography	Size and molecular weight	Simple operation, good reproductivity, large sample volumes, and realitively uniform size of the isolated exosomes	Low purity, further purification is required, and often used in combination with ultracentrifugation to improve efficency	Urine, serum, plasma, cerebrospinal fluid, and cell culture media	37, 39, 40, 41
Polymer‐based precipitation	Solubility and surface charge	Easy scalability, high productivity, user‐friendly, minimal labor needed, and commercial kits readily accessible	Low purity, contains free protein contamination, low recovery, and costly commercial kits	Urine, milk, blood, plasma, and serum	42, 43
Immunoisolation	Affinity	High purity, rapid, high specificity, and no effect on the structure and morphology	Costly, limited production, necessitates additional separation and purification procedures following antibody binding, and unsuitable for large‐scale applications	Urine, plasma, and cell culture media	44, 45, 46
Microfluidics‐based technologies	Acoustic, electrophoretic, electromagnetic affinity, density, and size	Rapid, high recovery rate, consumes small quantities of sample and reagent, and excellent portability	Not appropriate for extensive implementation, requires method validation and standardization, and is expensive	Blood, plasma, serum, lack of large‐scale studies, and not currently available for clinical use	47, 48, 49

### 2.1 Exosome isolation based on ultracentrifugation

Ultracentrifugation is a classical method using powerful centrifugal force to isolate exosomes. Exosomes aggregate and settle at the bottom of the ultracentrifuge tube due to centrifugal force. This method is categorized into differential ultracentrifugation and density gradient ultracentrifugation, depending on the separation principle employed (Figure 1B‐a).

#### 2.1.1 Differential ultracentrifugation

Differential ultracentrifugation is also known as ultracentrifugation. Ultracentrifugation is considered as the “gold standard” and one of the most commonly used and reported techniques for exosome extraction. The use rate of ultracentrifugation technology accounts for 56% of all exosome isolation technologies.^50^ The principle of ultracentrifugation is to isolate exosomes under different centrifugal forces according to the different densities and sizes of EVs in the bio‐sample.^51^ The initial step in ultracentrifugation involves eliminating particles that possess a high buoyancy density, including cells, cell debris, apoptotic bodies, and biopolymer aggregates. This is achieved through a series of centrifugation steps: first, centrifuging at 300–400*g* for 10 min to settle the majority of cells; then, at 2000*g* to eliminate cell debris; and finally, at 10,000*g* to separate aggregates of biopolymers, apoptotic bodies, and other structures with a buoyant density greater than that of EVs.^52^ Following this procedure, the ultimate sedimentation of exosomes is carried out through centrifugation of samples at a speed of 100,000–200,000*g* for a duration of 2 h or a minimum of 70 min.^53^

Ultracentrifugation is a physical separation method with little effect on the biochemical components of exosomes. It is easy to use and suitable for isolating and extracting exosomes from large‐volume samples; however, it is not appropriate for studying trace and valuable samples. In addition, the presence of contaminants, the long time required, the expensive equipment, and the large sample dose limit the effectiveness and use of ultracentrifugation in clinical trials and diagnostics. Exosomes can be damaged by excessively high rotational speeds, leading to a decrease in the activity of their contents such as RNA and DNA, used for subsequent analysis.

#### 2.1.2 Density gradient centrifugation method

Density gradient centrifugation is an improved method of traditional differential ultracentrifugation. It introduces an inert centrifugal medium in the centrifugal system, allowing exosomes to be transferred to the appropriate density gradient interval through ultracentrifugation. The medium of density gradient centrifugation is mainly sucrose and iodixanol.

The sucrose density gradient centrifugation method is to form a continuously distributed density gradient layer from low to high under the action of the centrifugal force. The exosomes are isolated by bands formed in different regions and concentrated in the density range of 1.13–1.19 g/mL. Gupta et al. compared differential ultracentrifugation and a modified method called one‐step sucrose cushion ultracentrifugation for isolating exosomes. They collected conditioned serum‐free media from human mesenchymal stem cells (MSCs) cultured for 48 h to isolate the exosomes. They removed cellular debris by centrifugation at 300*g* for 10 min, followed by centrifugation at 10,000*g* for 30 min to eliminate microvesicles. Then, under the condition of using equal volumes of pre‐processed conditioned media, exosome isolation was performed using both ultracentrifugation and one‐step sucrose cushion ultracentrifugation methods. A relatively high recovery of exosomes with cup‐shaped morphology was observed using the one‐step sucrose cushion ultracentrifugation method, as determined by nanoparticle tracking analysis (NTA) and transmission electron microscopy (TEM).^31^ Raj et al. conducted centrifugation at 400*g*, 800*g*, and 15,000*g* for 20 min at 4°C to eliminate cells and cellular debris. The supernatant was then subjected to ultracentrifugation at 200,000*g* for 1 h at 4°C to separate urinary vesicles. Following this two‐step differential centrifugation process, a subsequent double‐cushion sucrose/D_2_O centrifugation step was utilized to effectively isolate exosomes from the denser vesicles. This approach effectively isolated exosomes from the denser vesicles, enabling the solubilization and removal of major impurities associated with urinary exosomes.^32^ When compared to the sucrose density gradient centrifugation method, the iodixanol density gradient centrifugation method offers several advantages, including lower viscosity, metabolic inertness, nontoxicity to cells, and better preservation of cell integrity and functionality. Li et al. introduced a robust method platform called cushioned‐density gradient ultracentrifugation. They used a 60% iodixanol cushion for concentrating nanoparticles to enhance exosome recovery and maintain their physical integrity and biological activity. This cushion prevented pellet formation in the centrifuge tube. Additionally, exosome purification through density gradient ultracentrifugation effectively eliminated protein contaminants and non‐exosome nanoparticles. Moreover, iodixanol exhibited high biological inertness and compatibility with various downstream in vitro functional assays. It is also deemed suitable for animal studies, making it an excellent choice for reliable and efficient biochemical and physiological investigations of purified exosomes, eliminating the need for their removal.^54^ Konadu et al. devised a method to isolate exosomes from human immunodeficiency virus 1 (HIV‐1) particles in the plasma of infected individuals using iodixanol velocity gradients. Despite their comparable size and density, exosomes were found to separate into the upper fractions of the iodixanol gradients, characterized by lower density, while viral particles were observed in the lower fractions with higher density.^33^

Density gradient centrifugation enhances the purity of isolated exosomes using samples obtained via ultracentrifugation.^34^ However, this method is complicated to operate, requires high technical proficiency from the operator, has low throughput, is time‐consuming, and is not effective in removing lipoproteins and chylomicrons from blood samples.

### 2.2 Size‐based isolation method

The method of isolating exosomes based on their size has also been investigated. Ultrafiltration and size exclusion chromatography (SEC) isolate exosomes from other components in biological samples based on sizes.

#### 2.2.1 Ultrafiltration

Ultrafiltration is a technique that employs the pressure disparity across an ultrafiltration membrane to segregate exosomes according to their characteristic size. Ultrafiltration and membrane filtration are essentially synonymous (Figure 1B–b). Various ultrafiltration methods can effectively separate exosomes, including sequential filtration, tandem filtration, centrifugal ultrafiltration, and tangential flow filtration (TFF).^55^ These methods employ membrane filters with specific molecular weight or size exclusion limits, separating the suspended particles or polymers based on their size or molecular weight.

Syringe‐based sequential and tandem filtration are dead‐end techniques. Sequential filtration involves multiple rounds of filtration, with each round using a different molecular weight cutoff. On the contrary, the tandem filtration method combines multiple filters within a single syringe. In this method, the exosomes, which typically have a size exclusion threshold ranging from 20 to 200 nm, are trapped within an intermediate membrane. Centrifugal ultrafiltration, which integrates dead‐end filtration and centrifugation, enables the isolation of exosomes using nanoscale pores. A nanoporous membrane is rotated within a tube using centrifugal force to facilitate the passage of sample content. Before centrifugal ultrafiltration, centrifugation or dead‐end filtration is commonly conducted at 0.22 μm to remove larger particles such as cells, cell debris, and protein aggregates, preventing blockages.^56^ Recently, TFF has emerged as a novel method for isolating exosomes. Unlike traditional approaches, TFF operates by passing samples tangentially to the membrane, rather than applying pressure orthogonal to it. This unique mechanism prevents membrane blockages caused by particle accumulation, ensuring efficient filtration. Moreover, TFF offers several advantages over ultracentrifugation techniques. It enables the processing of larger fluid volumes with enhanced reproducibility and is known for its gentle treatment of samples. However, TFF typically requires more time than other filtration methods.^57^ Ultrafiltration methods are commonly combined with ultracentrifugation to isolate EVs from cells and proteins. Xu et al. developed a sequential centrifugal ultrafiltration (SCUF) method to investigate different subtypes of EVs released in vitro into the cell culture medium. They used the human colon carcinoma cell line LIM1863 as a model system and performed SCUF using hydrophilic polyvinylidene fluoride (PVDF) membranes with low protein‐binding properties (Millipore Durapore™ Ultrafree‐CL filters with pore sizes of 0.1, 0.22, 0.45, and 0.65 μm). This SCUF method is potentially scaled up using TFF, allowing for comprehensive functional studies of EV subtypes using various cell types and functional assays in the future.^58^

Ultrafiltration of exosomes is simpler, faster, and does not require specialized equipment compared with ultracentrifugation extraction. As the ultrafiltration process is carried out at room temperature and does not require the addition of chemical reagents, basically no component damage is caused to the exosomes, and it does not cause chemical pollution. The disadvantage is that membrane adhesion reduces the yield of exosomes, the pressure and shear force during filtration may cause the exosomes to deform and damage, and the filter membrane is easily damaged or blocked to influence the separation effect.

#### 2.2.2 Size exclusion chromatography

The principle of SEC is based on different sizes. The sample flows through the column, and substances larger than the pore size of the gel particles cannot enter the pores. They are eluted through the space between the porous gel and the mobile phase. However, small molecules remain trapped in the gel pores and are eventually eluted by the mobile phase, requiring a longer elution time (Figure 1B–c). The stationary phase or column can be packed with various gel polymers, including cross‐linked dextran (Sephadex), agarose, polyacrylamide (Biogel P), or allyl dextran (Sephacryl).^53^

To date, the SEC method has been successfully used for the isolation, purification, and enrichment of exosomes from a wide range of biological fluids, for example, urine,^59^ saliva,^60^ serum,^61^ and so on. The EVs in the supernatant of tumor cells consist of different types of vesicles, including exosomes, microvesicles, and apoptotic bodies. To distinguish tumor‐derived exosomes (TEX) from non‐TEX, researchers need to categorize the EVs into subgroups. This can be achieved by employing specific tumor cell culture conditions and using mini‐SEC.^39^, ^62^ Additionally, tissue exosomes, for example, from synovial tissue, can be extracted using SEC.^63^ SEC has become the preferred method for rapidly isolating relatively pure exosomes from plasma.^64^ Hong and colleagues successfully modified the SEC approach, which allows for simple and reproducible isolation from small plasma volumes (1 mL) of exosomes retaining the structural integrity and functional activity.^65^, ^66^

A majority of current reports on exosome isolation by size exclusion methods include a combination of SEC isolation with other isolation techniques. Yong et al. presented an approach that involved the preprocessing of plasma, combined with ultracentrifugation and SEC to isolate EVs and subsequently enrich exosomes, which enabled the use of a greater starting volume for exosome isolation, was highly reproducible and time‐efficient, and provided a greater yield.^67^ The combination of ultrafiltration and SEC led to a significant enhancement in the exosome quantity and concentration compared with ultracentrifugation. Specifically, it resulted in a maximum 58‐fold increase in the number of exosomes and a maximum 836‐fold decrease in the concentration of co‐purified soluble factors, taking into account the exosome yield.^68^ Yang et al. used Sepharose‐4B to construct a custom SEC column for separating and characterizing exosomes. The self‐made SEC column effectively segregated EVs from complex serum proteins, with EVs predominantly concentrated in fractions 8–13, exhibiting favorable morphology and yield. SEC proved to be superior in all aspects through a comprehensive comparison with the commonly employed ultracentrifugation and total exosome isolation commercial kit (Total Exosomes Isolation Reagent, TEI, 4478360, Invitrogen), striking a balance between isolation purity and yield.^69^ Nevertheless, the SEC method alone still possessed certain limitations and residual impurities. And when they used the SEC + ultracentrifugation approach ingeniously addressed the drawbacks of SEC and optimized the quality and purity of EVs derived from serum, surpassing the efficacy of either method used individually.^69^ Studies found that a more effective method could be achieved in terms of both efficiency and purity by implementing a modified serum sEVs isolation protocol, which involved two ultracentrifugation cycles and a 30% sucrose buffer.^34^

The advantages of SEC are its simple operation, good reproducibility, large sample size, and relatively uniform size of the isolated exosomes. The disadvantage is that it can isolate particles similar in size to others, resulting in reduced purity.^53^ Currently, commercial columns have been developed for isolating and purifying exosomes based on the principles of SEC.^70^, ^71^ The commercial product qEV column of iZON based on the SEC principle can extract exosomes with high purity from 150 μL to 10 mL of initial samples within 15 min, which ensures the stability of biochemical components and morphological structure of exosomes while extracting efficiently (http://www.moerybio.net/Products‐34788885.html).

### 2.3 Separation technology based on polymer precipitation

More than half a century ago, the polymer precipitation method was used to enrich and purify viruses.^72^ This method was also used to extract exosomes due to the similar size and biochemical properties of exosomes and virus particles.^73^ The principle of polymer coprecipitation technology is that the hydrophilic polymer interacts with the hydrophilic bond of the exocrine body of the sample to form a hydrophobic microenvironment around the exocrine body so as to form a precipitate and extract exosomes (Figure 1B–d).^26^ The exosome precipitation isolation method isolates exosomes by introducing chemical reagents (mainly polyethylene glycol, lectin, and so forth) to change exosome dispersibility or water solubility.

Polyethylene glycol (PEG) is the most commonly used polymer for exosome isolation. It effectively promotes the enrichment and production of exosomes.^74^ Ludwig et al. developed and enhanced a precipitation method using PEG to concentrate EVs from cell culture supernatants. This method demonstrated that a significant amount of co‐precipitated molecules such as bovine serum albumin could be effectively removed by washing the PEG pellet and reprecipitating it through ultracentrifugation.^75^ The first stimuli‐mediated isolation system for exosomes was developed by incorporating a thermal‐responsive, reductant‐cleavable copolymer onto the membranes of exosomes. This system used a thermal‐responsive copolymer called poly *N*‐isopropylacrylamide‐co‐*N*‐acryloxysuccinimide (PNN), which was attached to the lipid bilayer of exosomes to modulate their surface wettability. When the temperature of the PNN‐labeled exosome samples exceeded the lower critical solution temperature (LCST = 31°C), the attached PNN underwent a phase transition from hydrophilic to hydrophobic, leading to the spontaneous aggregation of PNN‐labeled exosomes (PNN‐Exos). Furthermore, the PNN conjugate could be detached from the exosomal surfaces through reductant‐triggered S–S cleavage.^76^ Studies also found that when hydrochloride was introduced into milk to facilitate isoelectric precipitation, it effectively isolated and purified bovine milk exosomes. This significant advancement contributed to the progress of research on the health management of dairy cattle and the development of DDSs in human medicine.^77^

Precipitation‐based exosome isolation methods are most attractive for clinical research and most widely used for product formation on the market because of their simple operation and speed, no exosome damage, and low demand for additional equipment for separation. Over the past few years, the commercially available polymer‐based precipitation technique has gained popularity due to its time‐saving nature, potential for improved reproducibility, user‐friendly approach, and ability to yield high‐purity vesicles containing small RNAs. This method outperformed traditional centrifugation‐based techniques, which varied in effectiveness depending on the user.^78^, ^79^ Many commercial kits separate exosomes based on the principle of polymer precipitation. Although the precipitation method does not yield pure exosomes, which may contain substances that interfere with downstream experiments, it is still an effective way to achieve rapid enrichment and concentration of EVs in large‐volume samples, saving both time and cost.^80^ With the continuous deepening of research, the polymer precipitation method has been continuously upgraded and improved, and the extraction effect is greatly improved.

### 2.4 Separation technology based on immunoaffinity capture

A variety of specific proteins are found on the membrane of exosomes, such as the typical four tetraspanin proteins CD9, CD63, and CD81.^81^ This feature provides a new idea for separating and extracting exosomes: using antibodies or aptamers of exosomes on the membrane of the specific protein (antigen) to produce an immune response, facilitating the separation of exosomes. The principle of immunoaffinity technology is to separate and enrich exosomes by identifying specific proteins of exosomes (Figure 1B–e). Depending on the type of antibody substrate carrier, immune separation methods can be categorized into magnetic bead immune separation, chromatographic stationary phase separation, and other techniques.

#### 2.4.1 Magnetic bead immune separation

Magnetic particles, such as iron, nickel, neodymium, or magnetite, can be easily functionalized with biomolecules, such as antibodies. This modification enables the magnetic particles to specifically attach to exosomes, facilitating their extraction from a complex matrix using magnetic actuation. This approach helps eliminate interference caused by the biofluid matrix, allowing for the preconcentration of exosomes and enhancing the sensitivity of the detection process.^44^

Anion‐exchange (AE) magnetic beads have been extensively studied for their potential in exosome isolation because of their rich ion‐exchange capacity, high binding capacity, and fast magnetic response. Studies found that the AE‐based exosome isolation method using these magnetic beads yielded highly efficient recovery rates (>90%) and resulted in exosome samples with high purities (14.42 × 10^10^ particles/mg).^45^ Recently, Guo et al. developed a Strep‐tag II‐based immunomagnetic isolation (SIMI) system. This system used modified capture antibodies on magnetic nanoparticles, enabling specific and reversible recognition between Strep‐Tactin and Strep‐tag II. Compared with the gold standard ultracentrifugation method, the SIMI system extracted approximately 59% more exosomes from the 293T cell culture medium, while also offering a shorter isolation time, higher purity, and improved biological activity.^82^ A novel method was developed to isolate exosomes from serum in a precise and time‐dependent manner. This approach used magnetic beads for capturing exosomes and employed light‐activated elution. This was achieved by attaching a CD63 aptamer to the surface of the magnetic beads using a light‐sensitive nitrobenzene group. The excitation of ultraviolet light was controlled at around 365 nm, and exosomes were selectively released from the beads while preserving their structural and functional integrity.^83^

The use of immunomagnetic beads for exosome separation has become popular due to their high specificity and convenient magnetic separation properties. However, traditional spherical beads with smooth surfaces and rigid interface modifications often result in compromised exosome enrichment performance and a recovery process that is not biocompatible. Jia et al. drew inspiration from the structure of the hedgehog and incorporated a burr‐like structure into the fabrication of immunomagnetic beads. Then, they conjugated the exosome‐targeting antibody (anti‐CD63) onto these burr‐mimicking nano‐spikes using a redox‐cleavable PEG, which acted as an antifouling component. These modified beads were named immunomagnetic hedgehog particles (IMHPs). Under optimal working conditions (200 μg, 1 h, room temperature), IMHPs achieved a satisfactory enrichment of exosomes with a capture efficiency of 91.70%. A reducing agent called tris (2‐carboxyethyl) phosphine hydrochloride (TCEP) (20 mM, 30 min, 37°C) was used to cleave the disulfide bonds to release the captured exosomes, resulting in an effective release with an efficiency of up to 82.45%.^84^ Furthermore, the surface of TiO_2_ contains a Lewis acid site that exhibits a robust affinity for phosphate ions, enabling it to selectively bind to intact biofilms. Consequently, TiO_2_ is also extensively employed for the enrichment of biofilm‐like substances. The use of TiO_2_ microspheres for exosome separation demonstrates significantly superior efficiency compared with the conventional methods. This technique not only reduces sample processing time but also enhances separation specificity.^85^ A novel approach was introduced to isolate urinary exosomes by combining the selective interaction between exosomes and TiO_2_ with ultrafiltration. This method effectively reduced nonspecific protein adsorption and expedited sample preparation. Additionally, the isolated exosomes retained their structural integrity and could be easily lysed for subsequent analysis.^86^

#### 2.4.2 Chromatographic stationary phase separation

Despite the availability of numerous exosome isolation options, alternative techniques that offer improved throughput and purity are strongly desired. Chemical separation and processing platforms, similar to those used in high performance liquid chromatography (HPLC), present a potential alternative approach. Hydrophobic interaction chromatography (HIC) can be employed to extract exosomes from seminal plasma obtained through intrauterine insemination (IUI) treatments.^46^ In 2019, Huang et al. developed a protocol for the isolation and quantification of human urinary exosomes using HIC on a polyester (PET) capillary‐channeled polymer (C‐CP) fiber stationary phase.^87^ Wang and colleagues investigated the application of PET C‐CP fibers in an HIC protocol for extracting exosomes from a human plasma sample. Their preliminary findings indicated that their method enabled the isolation of exosomes with similar yields and size distributions but at a significantly faster rate compared with the conventional isolation techniques. Moreover, it effectively reduced the presence of accompanying proteins and other impurities. Additionally, the C‐CP HIC approach offered advantages over commercial exosome kits in terms of cost (at $5 per column) and reusability (approximately 30 times).^88^ A study conducted in 2021 employed C‐CP fiber micropipette tips in an HIC solid‐phase extraction process. This novel approach allowed quick (<15 min) and affordable (<$1 per tip) isolation at sample volumes and timeframes relevant to clinical applications.^89^

#### 2.4.3 Others

In addition to the methods of immunomagnetic bead separation and chromatographic solid‐phase separation based on immunoadsorption for exosome isolation, other methods based on the principle of immunoadsorption for exosome isolation have been reported. Lee et al. devised a cost‐effective and user‐friendly point‐of‐care testing platform, known as paper‐based enzyme‐linked immunosorbent assay (p‐ELISA), to target EVs/exosomes. They achieved this by employing the streptavidin agarose resin‐based immobilization (SARBI) technique. This innovative SARBI p‐ELISA held great potential for application in resource‐constrained regions/countries.^90^ Barati et al. devised a novel coaxial nanofiber configuration to achieve highly effective exosome isolation from bodily fluids. This configuration comprised a polycaprolactone polymer core and a sub‐10‐nm gelatin shell. The gelatin shell exhibited remarkable sensitivity to temperature fluctuations, enabling the efficient release of captured exosomes at the physiological temperature of 37°C. Moreover, the thin gelatin layer served to minimize contamination of the isolated exosomes. The electrospun nanofibrous membrane's interconnected micro‐pores offered a substantial surface area for immobilizing specific antibodies, thereby facilitating the efficient capture of exosomes.^91^

Immune capture separation has high specificity, high purity, and ease of use, while preserving the structure and morphology of exosomes. However, this method is limited to the isolation of exosomes with positive markers, resulting in low extraction efficiency. In addition, the activity of exosomes can be easily affected by pH and salt concentration, making it unsuitable for large‐scale exosome isolation. In addition, high cost and low production hinder its further development and use.

### 2.5 Separation technology based on microfluidic control

Microfluidics is a signal detection–based exosome extraction technology. Currently, microfluidic methods for exosome separation can be broadly categorized into two main techniques: affinity‐based (label‐based) and label‐free isolation methods (Figure 1B–f).

#### 2.5.1 Techniques for the label‐based isolation of exosomes

Labeling‐based techniques for exosome isolation, usually by immunocapture, isolate exosomes from the bio‐sample. Hisey et al. presented a novel herringbone‐grooved microfluidic device chemically modified with antibodies targeting general and cancer exosome membrane biomarkers, specifically CD9 and EpCAM (epithelial cell‐specific marker). This innovative device enabled the efficient isolation of exosomes from small quantities of high‐grade serous ovarian cancer serum.^92^ Tayebi et al. developed an immunoaffinity‐based method for exosome purification by specifically targeting a major protein marker (CD63) on exosomes, and this innovative method efficiently isolated and allowed visualization of exosomes enriched on the surface of microspheres. The design allowed accurate fluorescence quantification of individual microbeads by capturing the average fluorescence density of each bead, thereby effectively reducing optical interference from background noise. Additionally, using a large‐scale microfluidic single‐bead trapping device facilitated molecular analysis of exosomes through on‐chip elution and lysis of the purified sample, enabling downstream analysis. Further, this design allowed multiple surface modifications, enabling the simultaneous capture and analysis of multiple exosomes, facilitating multiplexed exosome capturing and analysis.^93^ The design enabled precise quantification of individual microbeads' fluorescence by capturing the average fluorescence density of each bead, effectively minimizing optical interference from background noise. Furthermore, a large‐scale microfluidic single‐bead trapping device was used, facilitating exosome molecular analysis. This was achieved through on‐chip elution and lysis of the purified sample, enabling downstream analysis. Additionally, this design supported multiple surface modifications, allowing for the simultaneous capture and analysis of multiple exosomes, thus enabling multiplexed exosome capturing and analysis.

Over the past few years, a team of researchers developed and introduced a microfluidic system called exosome isolation and detection (EXID). In the isolation chamber, exosomes were isolated using immune magnetic beads and marked with anti‐Programmed cell death 1 ligand 1(PD‐L1) fluorescence probes. Subsequently, the exosome‐labeled beads were guided into the analysis region and captured using micropillar arrays to measure the fluorescence of PD‐L1. The EXID system successfully achieved integrated on‐chip processing, eliminating the need for off‐chip operations. Leveraging the micropillar array, the system enabled single‐bead analysis, facilitating the detection of exosome heterogeneity. This groundbreaking technology held immense potential as a tool for personalized cancer diagnosis and immunotherapy.^94^ Yu et al. proposed a highly integrated exosome separation and detection (ExoSD) chip, which represented a significant advancement building upon previous research. This chip offered a rapid and efficient solution for isolating and detecting exosomes. The microfilter structure within the separation zone underwent optimization to achieve a high level of purity in exosome isolation. Furthermore, a comb‐like structure made of nickel was devised to amplify the magnetic force exerted on immunomagnetic nanoparticles, resulting in a significant increase in the recovery rate of exosomes. Within the detection zone, the isolated exosomes were specifically labeled using fluorescent antibodies and subsequently detected using a confocal fluorescence microscope. As a proof‐of‐concept demonstration for the ExoSD chip, exosomes with both high purity and high recovery rates were successfully isolated from cell culture supernatant at various flow rates (40, 60, 80, and 100 μL/min).^47^ Li et al. developed a novel method called the homogenous magneto‐fluorescent exosome (hMFEX) nanosensor, which combined magnetic isolation and enhanced fluorescence measurement. This integrated approach enabled rapid and on‐site analysis of TEXs. The hMFEX nanosensor exhibited high specificity and could detect TEXs across a dynamic range of five orders of magnitude. Additionally, it had a limit of detection of 6.56 × 10^4^ particles/μL.^95^

Despite the improved efficiency and purity achieved by label‐based exosome isolation methods, further advancements are needed to increase throughput and enable targeting of multiple EV populations with different types of labels. These techniques can be costly due to the expensive labeling procedures. Moreover, they are often complex and time‐consuming due to the intricate steps involved in the labeling preparation and the need for washing steps to isolate the labeled exosomes. Furthermore, the capturing molecules used may introduce sample contamination and alter the properties of exosomes, potentially leading to inaccurate results in subsequent characterization processes and compromising the reliability of downstream analysis in clinical applications.

#### 2.5.2 Techniques for label‐free isolation of exosomes

Size‐based exosome isolation strategies are often studied because they offer unmarked separation, ensure size uniformity, and minimize sample bias. Chen and colleagues introduced a novel ultrafiltration approach that involved using dual coupled harmonic oscillations within a dual membrane filter setup to generate transverse waves. This approach achieved clogless and ultrafast purification of exosomes, overcoming the limitations of other methods.^96^ Yang et al. presented a novel microfluidic device capable of modulating the membrane pore size using ion‐sputtered gold layers of varying thickness. This innovative approach enabled the effective isolation of exosomes from body fluids based on the pore size.^97^ Recently, Hua et al. designed a double TFF‐based microfluidic device to separate and enrich exosomes. This device used porous membranes with pore sizes of 200 and 30 nm to sort vesicles ranging from 30 to 200 nm, enabling the isolation and purification of exosomes from conventional sources such as cell culture supernatants and human sera.^98^ Zhao et al. introduced an innovative approach automated centrifugal microfluidic disk system integrated with functionalized membranes (Exo‐CMDS). This system aimed to isolate and enrich exosomes. Subsequently, the enriched exosomes were subjected to a novel aptamer fluorescence system (Exo‐AFS), to efficiently detect exosome surface proteins. Exo‐CMDS yields highly qualified results, with an optimal exosomal concentration of 5.1 × 10^9^ particles/mL from trace amounts of blood samples (<300 μL) in only 8 min through the centrifugal microfluidic disk system. This approach accomplished exosome isolation and purification in a single step, providing an accurate clinical diagnosis system for lung cancer.^48^

Separating the exosomes from other components in the blood requires a significant amount of time and effort due to their small volume and low buoyancy density. Alternating current electrokinetic (ACE) microarray chip devices have a significant potential to address the challenges of exosome isolation and subsequent analysis of associated RNA, DNA, and protein biomarkers. The DEP force from the ACE microarray separates nanoparticles from fluid based on their dielectric properties. Ibsen et al. demonstrated the ACE microarray chip's ability to rapidly isolate and recover glioblastoma exosomes from undiluted human plasma samples. This device functions with a minimal plasma sample (30–50 μL) and can concentrate the exosomes around the ACE microelectrodes within 15 min. A simple buffer wash removes most plasma materials, leaving the exosomes concentrated on the microelectrodes. The entire isolation process, including on‐chip fluorescence analysis, is only completed in under 30 min.^99^

Exosome separation efficiency has also been improved by combining two or more isolation strategies. Acoustic waves are known for their high precision and biocompatibility in manipulating cells and other bioparticles. However, current acoustic‐based isolation strategies can only process pre‐treated biological fluids. This limitation necessitates additional equipment and time, increasing the risk of sample loss. Furthermore, current acoustic separation strategies can only distinguish between two types of targets, making it challenging to isolate exosomes directly from complex multicomponent fluids, such as undiluted blood. Wang et al. developed an acoustofluidic platform (the fusion of acoustics and microfluidics) for size‐based isolation of salivary exosomes. They found that the platform consistently isolated secretions from saliva samples for use in downstream saliva secretion liquid biopsy applications, regardless of viscosity changes and collection methods.^100^ Wu et al. presented a label‐free and contact‐free exosome isolation method based on acoustofluidics, which integrated acoustics and microfluidics to isolate exosomes directly from whole blood. The acoustofluidic platform consisted of two modules: a microscale cell‐removal module designed to remove larger blood components and an exosome isolation module for subgroup separation of EVs. In the cell‐removal module, 110‐nm particles were successfully isolated from a mixture of micro‐ and nanosized particles, achieving a yield of more than 99%. In the exosome isolation module, a 98.4% purity of exosomes was achieved from the EVs mixture. The isolation of exosomes from whole blood was successfully achieved by seamlessly integrating the two acoustofluidic modules onto a compact chip, surpassing an impressive blood cell‐removal rate of more than 99.999%. This device allowed for the rapid, biocompatible, label‐free, contact‐free, and continuous‐flow isolation of exosomes. It provided a distinctive method to study the impact of exosomes on the development and advancement of human diseases. Moreover, this technology held promise for various applications such as health monitoring, medical diagnosis, targeted drug delivery, and personalized medicine.^101^

In summary, the conventional method for isolating exosomes requires a significant amount of sample, which can pose challenges when dealing with limited sample availability. Additionally, the isolation and detection of samples are processed independently, causing potential inefficiencies. Numerous procedures are involved, from the initial isolation and purification of exosomes to the final identification via morphology and molecular biology. Therefore, implementing an efficient and straightforward integrated method for ExoSD is crucial. By monitoring exosomes in real time, microfluidics can quickly isolate exosomes and diagnose early noninvasive diseases, demonstrating promising application prospects in improving recovery, reducing sample volume, and shortening treatment time.^102^ Label‐free isolation methods for exosomes have several advantages, including lower cost, simpler and faster processing, and greater reproducibility. Additionally, unlabeled isolation can reduce potential sample contamination and allow the separated exosomes to maintain their natural characteristics, which does not affect downstream analysis and better supports the development of exosome‐based applications.

## 3 A COMPARISON OF EXOSOME ISOLATION METHODS AND THEIR APPLICATION IN ISOLATING CNS EXOSOMES

Various isolation methods are available for different purposes and applications. However, despite the development of numerous methods for exosome isolation and purification, some limitations still exist that cannot meet all requirements, which should be considered when planning experiments. The comparison between different isolation methods for exosomes is depicted in Table 1. Different isolation methods might introduce variations in the yield, purity, and integrity.^103^ Therefore, considering the advantages and disadvantages of these methods is crucial for downstream research applications.^104^ When selecting, combining, and optimizing methods, considering the starting material, equipment availability, therapeutic use, administration route, and desired end product is essential to achieve high yield and purity for clinical applications. Combining different isolation methods may yield better isolation results than using a single method. Studies have combined multiple methods for isolation and purification to enhance the yield and purity of exosomes, improving the isolation efficiency and enrichment.^105^ During the 2013 International Society for EVs conference, scholars suggested combining ultrafiltration technology with SEC to enhance the extraction purity of exosomes.^106^

The pathogenesis of CNS diseases is multifaceted and can be categorized based on their underlying mechanisms. Neurodegenerative diseases, such as Alzheimer's disease (AD), Parkinson's disease (PD), and Huntington's disease, are characterized by progressive degeneration of neurons. On the contrary, autoimmune diseases, such as multiple sclerosis and immune‐mediated encephalitis, involve an abnormal immune response targeting the CNS. A reliable and reproducible method is necessary for isolating small EVs from the brain serum, cerebrospinal fluid (CSF), and other biofluid samples to comprehensively explore the physiological and pathological roles of exosomes in the brain. Various methods for isolating exosomes have been reported, however, when isolating exosomes from typical samples of patients with CNS (e.g., 0.5 mL) for analysis, only a limited number of reported methods could generate sufficient exosomes. In this study, we provided a summary and introduction of these successful methods used for isolating exosomes from samples of CNS origin (Table 2). Multiple centrifugation and ultracentrifugation steps could be used to isolate exosomes from the Glioblastoma multiforme (GBM) cell culture supernatant.^107^ In 2022, D'Acunzo et al. described a detailed protocol: an iodixanol‐based high‐resolution density gradient separation method. This technique allowed quantitative and reproducible analysis of brain EV subtypes in normal and pathological brain conditions, including neurodegenerative diseases such as AD and PD.^108^ Exosomes could also be isolated from the CSF of patients with amyotrophic lateral sclerosis and other neurological disorders using SEC.^109^ A well‐established immunoprecipitation assay using anti‐L1CAM‐coated beads could be used to purify exosomes from the plasma of patients with PD and healthy controls.^110^ The systematic review and meta‐analysis showed that the ExoQuick kit offered optimum isolation of exosomes for PD diagnosis.^111^ Table 2 shows that the isolation of CNS exosomes is currently mainly performed using more established commercial kit methods. Exosomes can also be isolated from brain tissue, which provide valuable and unique insights into the intricate workings of the brain. They offer data that cannot be obtained from exosomes isolated from conditioned media or body fluids.^131^

**TABLE 2:** Exosome isolation methods and their corresponding exosomal markers for the diagnosis of CNS diseases.

Diseases	Origins	Isolation method and reference	Biomarker	Upregulated	Downregulated	Species
Alzheimer's disease	Serum	Total Exosome Isolation kit (Invitrogen),^112^ ExoQuick (System Biosciences),^113^ Exosome Isolation Q3 kit for serum (Wayen Biotechnologies)^114^	SNAP‐25, miR‐193b, and miR‐30b‐5p	N	Y	Human
		Total Exosome Isolation kit (Invitrogen),^112^ Exosome Isolation Q3 kit for serum (Wayen Biotechnologies),^114^ ExoQuick (System Biosciences)^115^	miR‐34b, miR‐125b, miR‐130b, miR‐22‐3p, miR‐378a‐3p, miR‐135a, and miR‐384	Y	N	Human
	Plasma	ExoQuick Precipitation Solution kit (System Biosciences),^116^ ExoQuick Exosome Precipitation (SBI)^117^	HSP70, P‐S396‐tau, and Aβ1‐42	Y	N	Human
	Serum neuro‐exosomal	Sucrose step gradient^118^	NEX tau proteins	Y	N	Mice
	Blood neuro‐exosomal	ExoQuick Exosome Precipitation Solution kit (System Biosciences)^119^	GAP43, neurogranin, SNAP25, and synaptotagmin 1	N	Y	Human
	Cerebrospinal fluid	miRCURY™ Exosome Isolation Kit (Exiqon)^120^	miR‐451a and miR‐605‐5p	N	Y	Human
			miR‐125b‐5p	Y	N	Human
AD and aMCI	Blood neuronal	ExoQuick Exosome Precipitation Solution kit (System Biosciences)^121^	Aβ42, T‐tau, and P‐T181‐tau	Y	N	Human
Parkinson's disease	serum	Total Exosome Isolation Reagent (from other body fluids) (Invitrogen)^122^	miR‐19b	N	Y	Human
		Total Exosome Isolation Reagent (from other body fluids) (Invitrogen),^122^ immunological capture,^123^ ExoQuick (System Biosciences)^115^	α‐Synuclein, miR‐195, miR‐24, let‐7d, miR‐15b, miR‐24, miR‐142‐3p, miR‐181c, and miR‐222	Y	N	Human
	Plasma	PureExo® Exosome Isolation Kit (101Bio),^124^ ExoQuick	miR‐505	N	Y	Human
			miR‐331‐5p	Y	N	Human
	Blood neuro‐exosomal	Exosome Precipitation Solution kit (System Biosciences)^125^	DJ‐1 and α‐synuclein	Y	N	Human
	Cerebrospinal fluid	Sucrose density gradient centrifugation^126^	miR‐1 and miR‐19b‐3p	N	Y	Human
			miR‐153, miR‐409‐3p, miR‐10a‐5, and let‐7 g‐3p	Y	N	Human
	Saliva	XYCQ EV Enrichment kit^127^	α‐Synuclein	Y	N	Human
	Urine	Differential centrifugation^128^	Calbindin and SNAP23	Y	N	Human
Traumatic brain injury	Neuro‐exosomal	ExoQuick Plasma prep and Exosome precipitation kit (System Biosciences)^129^	Aβ42	Y	N	Human
	Saliva	Differential centrifugation^130^	CDC2, CSNK1A1, and CTSD	Y	N	Human
Frontotemporal Dementia	Plasma	ExoQuick Precipitation Solution kit (System Biosciences)^116^	HSP70	Y	N	Human

## 4 ADVANCEMENTS IN EXOSOME RESEARCH FOR THE DIAGNOSIS AND TREATMENT OF CNS DISEASES

In recent years, remarkable advancements have been made in studying exosomes and their role in intercellular communication. Exosomes have emerged as key players in regulating network connectivity and transport within the CNS. In microglial exosomes, the level of miR‐124‐3p significantly increased from the acute phase to the chronic phase of traumatic brain injury (TBI). The upregulation of miR‐124‐3p targets a sub‐family of the PDE4 family (PDE4B), inhibiting the activity of the Mammalian target of rapamycin (mTOR) signaling pathway, thereby suppressing neuronal inflammation and promoting neurite outgrowth.^132^ Exosomes derived from astrocytes have the potential to transport miR‐17‐5p, providing a protective shield for neonatal rats against hypoxic–ischemic brain damage (HIBD) by effectively inhibiting the expression of *Homo sapiens* BCL2 interacting protein 2 (BNIP2).^133^ In addition, specific components have been identified in exosomes, such as proteins, nucleic acids, and lipids, that can serve as indicators of disease progression or treatment response. By analyzing the composition and quantity of exosomes in biofluids such as CSF or blood, clinicians can gain valuable insights into the underlying pathology and monitor the effectiveness of therapies. Exosomes also have potential applications in CNS therapy. As a therapeutic agent or novel drug carrier, exosomes can cross the BBB and achieve effective drug delivery.^134^, ^135^ Figure 2 shows the overview of exosomes in the diagnosis and treatment of CNS diseases.

### 4.1 Application of exosomes in diagnosing neurological disorders

Using exosomes as potential biomarkers for CNS diseases is one of the main application areas. Exosomes have emerged as attractive targets for clinical diagnosis and biomarker discovery due to the following reasons. First, the contents of exosomes, including lipids, proteins, nucleic acids, and more, undergo changes during disease progression, potentially reflecting the disease status. Second, exosomes can be noninvasively isolated from easily accessible biological fluids such as blood, urine, and saliva. The noninvasive nature of exosome isolation is particularly important for early disease diagnosis, especially in the CNS. Third, exosomes possess a double‐layered membrane that protects potential biomarkers from degradation. Fourth, exosomes are highly stable, making them clinically feasible, as samples can be stored for extended periods before analysis. Fifth, exosomes carry markers associated with their cell of origin, allowing traceability. Finally, exosomes can cross the BBB, providing information about neural cells that would otherwise be difficult to obtain without invasive techniques.^136^

The blood is the only tissue in contact with all organs and carries a great deal of valuable information about the organism. Biomarkers derived from blood exosomes are highly sought after in the field of neurodegenerative diseases. Plasma exosomes were found to spread and cluster around β‐amyloid plaques in an animal model of AD.^137^ miR‐193b, miR‐135a, and miR‐384 from plasma exosomes are aberrantly expressed in AD and may serve as potential biomarkers for diagnosing AD.^112^ Serum exosomal miR‐19b, miR‐195, and miR‐24 are aberrantly expressed in PD and may serve as potential biomarkers for PD diagnosis.^122^ Neuronal exosomes are used as a new source of biomarkers for neurodegenerative diseases. Neuronal‐derived exosomal growth associated protein 43 (GAP43), neurogranin, synaptosome associated protein 25 (SNAP25), and synaptotagmin 1 serve as biomarkers that accurately reflect pathological changes in the AD brain and possess the ability to distinguish between AD and amnestic mild cognitive impairment (aMCI). Interestingly, when these biomarkers were combined, they could detect preclinical AD 5–7 years prior to the onset of cognitive impairment.^119^ The increased release of α‐synuclein serum neuronal exosomes precedes the diagnosis of PD, persists as the disease progresses, and is associated with the aggregation of proteins, predicting and distinguishing PD from atypical Parkinson's syndrome.^123^ The aforementioned studies have demonstrated the potential of CNS‐derived blood exosomes as a source of diagnostic, prognostic, and progression biomarkers for neurodegenerative diseases. In addition to blood products, exosomes from other biological fluids, such as CSF, saliva, and urine, have been used for diagnostic purposes. Exosomes have been detected in the CSF and cell culture media of humans as well as model species such as mice and monkeys.^138^ CSF exosomal RNA molecules are reliable biomarkers with fair robustness regarding specificity and sensitivity in differentiating PD from healthy and diseased (AD) controls. Aberrant expression of miR‐153, miR‐409‐3p, miR‐10a‐5, let‐7g‐3p, miR‐1, and miR‐19b‐3p in CSF‐derived exosomes has emerged as a potential biomarker for the diagnosis of PD.^126^ Collecting saliva from patients is less invasive than collecting blood, and saliva is easier to handle because it does not clot. However, saliva has been studied much less than plasma or serum as a source of biomarkers in neurodegenerative (and systemic) diseases, probably because of the higher variability and risk of contamination in saliva compared with blood. Rani et al. observed significant differences in the concentration of salivary exosomes among groups of patients with cognitive impairment and AD (*P* = 0.0023) compared with a healthy control cohort. They further demonstrated a novel method based on TEM technique to directly correlate the concentration of salivary exosomes with the progression of cognitive impairment in AD. This innovative approach proved to be a potentially cost‐effective screening method for early disease detection.^139^ In addition, aberrant expression of calbindin and SNAP23 in urinary exosomes was also identified as a potential biomarker for the diagnosis of PD.^128^ Table 2 summarizes the biomarkers used in exosomes from different sources for the diagnosis of CNS diseases and their corresponding methods of exosome isolation.

In summary, the aforementioned studies provided evidence for the potential of exosomes as biomarkers for CNS diseases; using exosomes as molecular diagnostic markers had promising applications. However, at the same time, some issues still need to be considered. The first consideration is the reliability of exosomes as CNS biomarkers. Only small amounts of pathogenic proteins cross the BBB and move to the periphery, and the levels of pathogenic proteins in the blood are influenced by many organs. Therefore, the levels of pathogenic proteins in plasma neuron–derived exosomes may not be sensitive to small pathological biological changes in the brain.^140^ The second consideration is the reliability and consistency of the experimental results. The principal steps of various exosome extraction methods are inconsistent, the exosome quantification methods applied are not uniform, and these problems may lead to poor reproducibility of the experiments. In conclusion, the research on exosomal biomarkers is still in its early stages, and it is hoped that these issues will be addressed in future studies, thus providing new support for the diagnosis.

### 4.2 Application of exosomes in treating neurological disorders

The transportation of large protein molecules is limited due to the restrictive nature of the BBB, resulting in the inability to use many drugs that can effectively treat various neurological disorders in clinical settings. However, exosomes are vesicles derived from cells and enclosed by a double‐layered lipid membrane, demonstrating the remarkable ability to traverse the BBB effectively.^141^ Exosomes possess the capacity to deliver their cargo directly to recipient cells. This feature makes them well‐suited as therapeutic agents, including engineered exosomes, which offer advantages that soluble factors alone cannot achieve. Moreover, exosome‐based treatments exhibit enhanced safety compared with cell‐based therapies involving therapeutic cell transplantation and offer regulatory advantages, further supporting their potential as a viable treatment option for neurological disorders.

#### 4.2.1 Cell‐free therapy based on stem cell‐derived exosomes

Exosomes themselves can be used as a therapeutic approach for CNS diseases.^142^ Exosomes derived from MSCs (MSC‐EVs) can cross the BBB at the tissue level and promote fusion with the cell membrane, allowing the direct release of their contents into the cytoplasm. This mechanism effectively avoids capture by endosomes/lysosomes and enables the exosomes to traverse organelle membrane barriers.^143^ MSCs are better producers of EVs than other kinds of cells and possess various biological functions.^144^ The potential therapeutic approaches of MSC‐EVs for neurodegenerative diseases are as follows: enhanced neurofunctional recovery,^145^ remyelination,^146^ synaptic plasticity,^147^ and neuroprotection.^148^ In addition, injections of MSC‐derived EVs have been reported to enhance the resistance of hippocampal neurons to the detrimental effects of oxidative stress and β‐amyloid (Aβ)‐induced synaptic damage.^149^ These functions play a multifaceted role in regulating the pathophysiological processes of CNS diseases, making MSC‐EVs a promising therapeutic strategy for improving neurological function recovery.

Bone marrow MSC–derived exosomes (BMSC‐exos) administered through intracerebroventricular injection into a mouse model of AD induced by streptozotocin demonstrated that BMSC‐exos could improve AD‐like behavioral manifestations in mice. This improvement may be associated with the modulation of glial cell activation by BMSC‐exos and the neuroinflammatory and hippocampal BDNF‐related neuropathological changes.^150^ MSC‐derived exosomes have been successfully used as a potential therapy to combat the progression of PD and improve symptoms, resulting in similar therapeutic effects as MSC transplantation.^151^ Li et al. discovered that exosomes derived from MSCs carrying miR‐188‐3p inhibited the expression of inflammasomes and autophagosomes by targeting the 3' Untranslated Regions (UTR) of Nucleotide‐ binding oligomerization domain, leucine‐ rich repeat and pyrin domain‐ containing 3 (NLRP3) and cyclin‐dependent kinase 5, thereby alleviating nigral damage in a 1‐methyl‐4‐phenyl‐1,2,3,6‐tetrahy dropyridine‐induced PD mouse model.^152^ Chen et al. demonstrated that the exosomes secreted by human umbilical cord MSCs reached the substantia nigra via the BBB, alleviated apomorphine‐induced asymmetric rotation, reduced nigral dopaminergic neuron loss and apoptosis, and upregulated dopamine levels in the striatum, which could treat PD.^153^ Exosomes derived from dental pulp stem cells alleviated neuroinflammation in middle cerebral artery occlusion (MCAO) mice by inhibiting the high‐mobility group box 1/toll‐like receptor 4/myeloid differentiation factor 88/nuclear factor‐κB signaling pathway, thereby reducing ischemic brain injury.^154^ Guo et al. found that BMSC‐exos alleviated apoptosis of hippocampal neurons in rats and improved symptoms in a rat model of depression by upregulating the expression of miR‐26a.^155^ Moreover, exosomes from human BMSCs may be used as a safe noninvasive treatment to ameliorate behavioral symptoms of patients with autism spectrum disorders.^156^

#### 4.2.2 Engineered exosome therapy

MSC‐EVs have limitations in their therapeutic and diagnostic capabilities for CNS diseases and may not fully meet treatment requirements. However, fortunately, exosomes can be modified and engineered to serve as carriers for treating CNS diseases. Currently, several methods are available for loading drugs (small‐molecule drugs, transmembrane proteins, and nucleic acid drugs) into exosomes, including sonication, electroporation, transfection, incubation, extrusion, saponin‐assisted loading, transgenesis, freeze–thaw cycles, thermal shock, pH gradient method, and hypotonic dialysis. All drug‐loading techniques have their advantages and disadvantages.^157^ Using advanced engineering techniques to engineer exosomes has improved the efficacy of exosomes as agents for disease treatment.

In the field of AD treatment, engineered exosomes have been shown to have a pivotal role. AD is clinically characterized by progressive dementia caused by the pathological accumulation of neurofibrillary tangles and Aβ plaques. Notably, tau hyperphosphorylation and Aβ hyperaggregation have been identified as two crucial features in the AD brain, even occurring decades before the onset of cognitive dysfunction.^17^ Studies showed that beta‐site APP‐cleaving enzyme 1 (BACE1) was a protease responsible for the N‐terminal cleavage of the amyloid precursor protein, which generated the β‐amyloid peptide. When exosomes were designed to express neuron‐targeting peptides on their surface and injected into the blood of mice after loading siRNA by electroporation, they exhibited a significant ability to cross the BBB, resulting in a significant reduction in the BACE1 expression.^158^ Moreover, targeting the phosphorylation of the tau protein has emerged as a crucial therapeutic strategy for AD aggregation therapy. Curcumin, a natural compound derived from a plant source, has shown promise as a potential drug for AD treatment by effectively inhibiting the phosphorylation of the tau protein. However, one of the challenges faced by curcumin, similar to many other drugs, is its limited ability to cross the BBB effectively. To address this challenge, a team led by Wang et al. conducted research and developed a formulation called Exo‐cur, which incorporated curcumin. This innovative formulation was engineered to efficiently penetrate the BBB, reach brain tissue, and inhibit tau phosphorylation. It showed great potential in targeted drug delivery for AD treatment and neurological recovery.^159^ Similarly, quercetin (Que) has shown potential in preventing tau pathology and providing neuroprotection, leading to improvements in cognitive and functional symptoms associated with AD. However, the clinical application of Que has been hindered by its limited brain targeting and low bioavailability. Qi et al. developed exosomes loaded with quercetin (Exo‐Que) to enhance the drug's bioavailability and its targeting to the brain. The findings revealed that Exo‐Que demonstrated superior efficacy compared with free quercetin in alleviating the symptoms of AD.^160^

PD is the second most common neurodegenerative disorder of CNS after AD. The self‐assembled nano carrier PR‐EXO/PP@Cur, developed by Peng et al., enables intranasal delivery of curcumin loaded with exosomes overcame the limitations of MSC‐EVs in effectively clearing neurotoxic α‐syn aggregates and targeting dopaminergic neurons. Additionally, it addressed the challenges of curcumin crossing the BBB. This innovative approach enabled a three‐pronged synergistic treatment for PD.^143^ Qu et al. developed an innovative approach involving the loading of exosomes with dopamine and administering them systemically to a mouse model of PD to combat PD. The results demonstrated remarkable therapeutic effects, highlighting the potential of this novel drug delivery method in treating PD.^161^ Kojima et al. reported a set of devices called EXOsomal transfer into cells (EXOtic) that could efficiently and customarily produce designer exosomes in engineered mammalian cells. These genetically encoded devices enhanced exosome production, enabled specific mRNA packaging, and delivered mRNA into the cytoplasm of target cells, facilitating efficient intercellular communication without the need for exosome concentration. Furthermore, the EXOtic device had potential utility in RNA‐based therapeutic applications; designer exosomes could alleviate neurotoxicity and neuroinflammation in both in vitro and in vivo PD models.^162^

Except for AD and PD, engineered exosomes also have better therapeutic effects for other CNS diseases. Engineered exosomes loaded with neurotrophic factors and modified with rabies virus glycoprotein (RVG) ligand peptides could selectively target the damaged cerebral cortex in ischemic stroke, alleviating post‐stroke inflammation and promoting neuronal survival through the action of nerve growth factor (NGF).^163^ Exosome‐loaded resveratrol formulations for treating multiple sclerosis, which effectively suppressed inflammation in both the CNS and peripheral system, led to significant improvements in the clinical progression of diseases.^164^ In zebrafish, the incubation method enabled the loading of exosomes with doxorubicin and paclitaxel, allowing them to cross the BBB for brain cancer treatment. When administered separately, both drugs showed no absorption by the brain.^165^ In addition, mice treated with Exo‐cur or exosomes loaded with signal transducer and activator of transcription 3 (Stat3) inhibitor JSI124 (Exo‐JSI124) via intranasal administration were protected against lipopolysaccharide‐induced encephalitis and experimental autoimmune encephalomyelitis induced by myelin oligodendrocyte glycoprotein peptide and exhibited delayed brain tumor growth in the Glioma 26 (GL26) tumor model.^166^

## 5 DISCUSSION

Exosomes, as a widely existing bioactive substance involved in intercellular information transmission, play a vital role in physiological and pathological processes. As an emerging field of research in recent years, the classification, generation mechanism, and characteristics of exosomes have also been gradually recognized. However, the separation and identification of exosomes is difficult due to their small size and low density, which is an important factor restricting research and transformation. Establishing a mature, stable, convenient, and fast method for the separation of exosomes is a premise for their use in medical diagnosis, prediction, and the pharmaceutical field. Many kinds of exosome isolation techniques have been developed. Each separation method is based on a unique property of exosomes, such as size, density, compressibility, surface protein, and so forth. However, these separation techniques still have problems such as low recovery, low purity, and possible contamination, seriously affecting the analysis of downstream results. The selection of methods for extracting exosomes from body fluids should be based on the characteristics of body fluid types, sample volume, follow‐up research needs, and laboratory conditions. At the same time, rather than relying on a single method for extraction, using a combination of different methods to leverage complementary advantages is currently a wiser choice.

Moreover, the study of exosomes in CNS diseases is an exciting and rapidly evolving field. Exosomes, derived from host cells, CSF, blood, and so on, contain DNA, RNA, proteins, and other molecules, providing insights into host cell attributes. Consequently, exosomes are promising indicators for the early detection of neurological ailments. Scientists can obtain information aiding in the early identification and detection of neurological disorders by analyzing exosome contents. Additionally, MSC‐EVs show promise as personalized medicines for neurological disorders. However, their efficiency, homogeneity, and stability need to be ensured before they can be established as effective therapeutic tools. Not all EVs derived from MSCs are equivalent.^167^ Several strategies are necessary to enhance the potential therapeutic effects of MSC‐EVs, such as,: (I) developing platforms for large‐scale, cost‐effective production of MSC‐EVs to meet clinical needs; (II) improving detection methods for MSC‐EVs to determine the optimal dosage, frequency, and tissue distribution; and (III) enhancing modified strategies of MSC‐EVs to improve their biological functions and target site accumulation.^168^ Exosomes as DDSs offer a range of advantages: biocompatibility, biodegradability, intercellular communication capabilities, targeted delivery, immunomodulatory properties, low immunogenicity, low toxicity, and potential for personalized medicine. Among these, the potential for personalized medicine is an exciting aspect of exosomes. They can be isolated from specific cell types, enabling tailored cargo and targeting strategies that align with individual patient needs. This personalized approach holds great promise for advancing precision medicine. These attributes position exosomes as a promising avenue for innovative therapeutic interventions. However, currently no completed or published clinical trials have reported on the role of exosomes in treating CNS diseases; but several clinical trials and studies that use exosomes as biomarkers are ongoing, which are worth noting. For instance, using miRNA‐manipulated microglial exosomes shows potential as a novel therapy for TBI and other neurologic diseases. Specifically, miR‐124‐3p holds promise as a therapeutic target for mitigating neuronal inflammation following TBI.^132^ Exosomes as DDSs targeted miR‐210 through exosomes for angiogenic therapy following cerebral ischemia in mice.^169^ Evox Therapeutics, under the leadership of Tony De Fougerolles, established strategic collaborations in 2020 with industry leaders such as Takeda and Eli Lilly. These partnerships focused on harnessing the potential of exosome‐based therapies to address unmet medical needs in the fields of rare diseases and neurological disorders, respectively.^170^

In summary, the isolation of exosome has yet to find a widely recognized and efficient method. The isolation technology and subsequent identification methods of exosomes still need continuous improvement. Exosome research and application has two main technical challenges: simplifying extraction and increasing production, and developing effective methods to distinguish exosomes from other EVs, especially functional vesicles. This study comprehensively analyzed the current research progress in exosome isolation strategies. It thoroughly introduced the existing technologies for exosome isolation and offered insights into the development of new methods for efficiently isolating exosomes from various bio‐sample. Exosomes hold great potential for improving diagnosis, monitoring disease progression, and developing innovative therapeutic strategies, with promising applications in clinical diagnostics. However, some issues still need to be resolved in future research. More advanced separation techniques are needed for exosome identification and mass production. The mechanisms by which exosomes regulate the CNS are not fully understood. Moreover, surface modification and drug loading of exosomes remain a challenge.

## CONFLICT OF INTEREST STATEMENT

The authors declare that there are no conflicts of interest.

## Data Availability

Data sharing not applicable to this article as no datasets were generated or analysed during the current study.
